# Characterization of Genetic Heterogeneity in Recurrent Metastases of Renal Cell Carcinoma

**DOI:** 10.3390/cancers13246221

**Published:** 2021-12-10

**Authors:** Carolin Sauter-Meyerhoff, Regina Bohnert, Pascale Mazzola, Viktoria Stühler, Siarhei Kandabarau, Florian A. Büttner, Stefan Winter, Lisa Herrmann, Steffen Rausch, Jörg Hennenlotter, Falko Fend, Marcus Scharpf, Arnulf Stenzl, Stephan Ossowski, Jens Bedke, Matthias Schwab, Elke Schaeffeler

**Affiliations:** 1 Dr. Margarete Fischer-Bosch Institute of Clinical Pharmacology, 70376 Stuttgart, Germany; carolin.meyerhoff@ikp-stuttgart.de (C.S.-M.); regina.bohnert@ikp-stuttgart.de (R.B.); pascale.mazzola@med.uni-tuebingen.de (P.M.); siarhei.kandabarau@ikp-stuttgart.de (S.K.); florian.buettner@ikp-stuttgart.de (F.A.B.); stefan.winter@ikp-stuttgart.de (S.W.); elke.schaeffeler@ikp-stuttgart.de (E.S.); 2 Department of Urology, University Hospital Tuebingen, 72076 Tuebingen, Germany; viktoria.stuehler@med.uni-tuebingen.de (V.S.); lisa.herrmann@student.uni-tuebingen.de (L.H.); steffen.rausch@med.uni-tuebingen.de (S.R.); joerg.hennenlotter@med.uni-tuebingen.de (J.H.); arnulf.stenzl@med.uni-tuebingen.de (A.S.); jens.bedke@med.uni-tuebingen.de (J.B.); 3 Institute of Pathology and Neuropathology, University Hospital Tuebingen, 72076 Tuebingen, Germany; falko.fend@med.uni-tuebingen.de (F.F.); marcus.scharpf@med.uni-tuebingen.de (M.S.); 4 Institute of Medical Genetics and Applied Genomics, University of Tuebingen, 72076 Tuebingen, Germany; stephan.ossowski@med.uni-tuebingen.de; 5 German Cancer Consortium (DKTK), Partner Site Tuebingen, German Cancer Research Center (DKFZ), 69120 Heidelberg, Germany; 6 Departments of Clinical Pharmacology, Pharmacy and Biochemistry, University of Tuebingen, 72076 Tuebingen, Germany; 7 Cluster of Excellence iFIT (EXC2180) “Image-Guided and Functionally Instructed Tumor Therapies”, University of Tuebingen, 72076 Tuebingen, Germany

**Keywords:** renal cell carcinoma, metastasis, next-generation sequencing, pharmacogenomics, personalized therapy

## Abstract

**Simple Summary:**

Survival rates in metastatic renal cell carcinoma (RCC) are still low despite novel therapies available. Thus, knowledge of molecular characteristics of distant metastases is important for personalized treatment strategies. Therefore, we investigated the genetic landscape of metastases, including synchronous and/or recurrent metastases to elucidate potential drug target genes and clinically relevant mutations. Furthermore, differences in mutational composition in different metastatic sites and over the course of the disease and treatment will demonstrate the importance of somatic profiling for precision medicine in RCC, thereby improving disease management in the future.

**Abstract:**

Metastatic renal cell carcinoma (RCC) exhibits poor prognosis. Better knowledge of distant metastases is crucial to foster personalized treatment strategies. Here, we aimed to investigate the genetic landscape of metastases, including synchronous and/or recurrent metastases to elucidate potential drug target genes and clinically relevant mutations in a real-world setting of patients. We assessed 81 metastases from 56 RCC patients, including synchronous and/or recurrent metastases of 19 patients. Samples were analysed through next-generation sequencing with a high coverage (~1000× mean coverage). We therefore established a novel sequencing panel comprising 32 genes with impact on RCC development. We observed a high frequency of mutations in known RCC driver genes (e.g., >40% carriers of *VHL* and *PBRM1* mutations) in metastases irrespective of the metastatic site. The somatic mutational composition was significantly associated with cancer-specific survival (*p*(logrank) = 0.03). Moreover, we identified in 34 patients at least one drug target gene as well as clinically relevant mutations listed in the VICC Meta-Knowledgebase in 7%. In addition to significantly higher mutational burden in recurrent metastases compared to earlier ones, synchronous and/or recurrent metastases of individual patients, even after a time-period >2 yrs, shared a high proportion of somatic events. Our data demonstrate the importance of somatic profiling in metastases for precision medicine in RCC.

## 1. Introduction

Renal cell carcinoma (RCC) is among the ten most frequently diagnosed cancers worldwide [1]. Metastatic disease is present in ~30% of clear cell RCC (ccRCC), the most common subtype of sporadic RCC, and correlates with poor survival rates even in case of targeted or immunotherapy [2,3,4,5]. Primary ccRCC tumours are characterized by genomic aberrations in the tumour suppressor gene *VHL* as well as variations in other driver genes such as *BAP1, PBRM1,* and *SETD2* [6,7,8]. Moreover, profound intra-tumoral heterogeneity (ITH) was reported in RCC [9,10]. As proposed by Turajlic et al., [10] progression of RCC is majorly influenced by the somatic mutational composition of tumours. Based on results from multiregional sequencing, different evolutionary ccRCC subtypes could be defined [11]. Besides these important genetic determinants of a patient’s outcome, transcriptome analysis of primary RCC resulted in different gene expression scores [12,13,14,15,16,17] for prediction of worse outcomes.

Thus far, most studies investigated the genetic landscape of primary tumours [18,19]. As poor survival in RCC patients is especially associated with metastatic disease, there is a need for better understanding of underlying molecular mechanisms in metastases, which are altered by mutational evolutionary processes and selective treatment pressure. Generally, several theories exist regarding the development of metastasis, involving, for example, tumour microenvironment or epithelial-mesenchymal transition (EMT) [20,21]. In contrast to primary tumours, genetic variation in RCC metastases [22] and especially in multiple metastases of an individual patient in the same or in different organs have been less well-studied. Thus, our aim was to not only investigate ccRCC-derived metastases in different metastatic sites, but also to study the genetic heterogeneity in different metastases from one individual using next-generation sequencing. The observed genetic variation was correlated with clinical outcome data. Thereby, we provide further insight into genetic variation occurring in RCC-derived metastases, particularly in recurrent metastases of individual patients over time and after systemic therapy.

## 2. Materials and Methods

### 2.1. Patient Cohort

The study cohort comprises 56 patients treated at the Department of Urology, University Hospital Tuebingen, Tuebingen, Germany. In total, 81 formalin fixed and paraffin embedded (FFPE) metastasis samples were collected after surgical intervention. Multiple metastases, including 6 matched primary tumours were obtained from 19 patients. The study was approved by the ethics committee of the University of Tuebingen, Germany and informed written consent was provided by each subject prior to surgical resection. Further information about patients’ characteristics and collected metastasis samples is given in Table S1.

### 2.2. Next Generation Sequencing (NGS)

For further details on NGS sample preparation and data analyses, see supplementary material. In brief, NGS library preparation was performed using the TruSeq Custom Amplicon Low Input Library Prep Kit (Illumina, San Diego, CA, USA) and our newly established gene panel (DesignStudio, Illumina, San Diego, CA, USA) targeting regions of 32 different genes that are already known to play an important role in the development and progression of RCC. Sequencing was performed on a MiniSeq platform (Illumina, San Diego, CA, USA). 

### 2.3. Statistical Analyses 

Statistical analyses were performed in R (version 3.6.1) using additional packages from CRAN (http://cran.r-project.org) and from the Bioconductor software project (http://www.bioconductor.org, version 3.11): survival (version 3.2.7, accessed on 21 May 2020) [23] and survminer (version 0.4.8, accessed on 25-07-2020) [24]. Construction and visualization of phylogenetic trees with annotations was performed using MesKit 1.0.1 [25] in R (version 4.0.3). Further details of statistical analyses are given in supplementary data. 

## 3. Results

### 3.1. Somatic Variants of RCC Metastases in Different Organs

**Figure 1:**
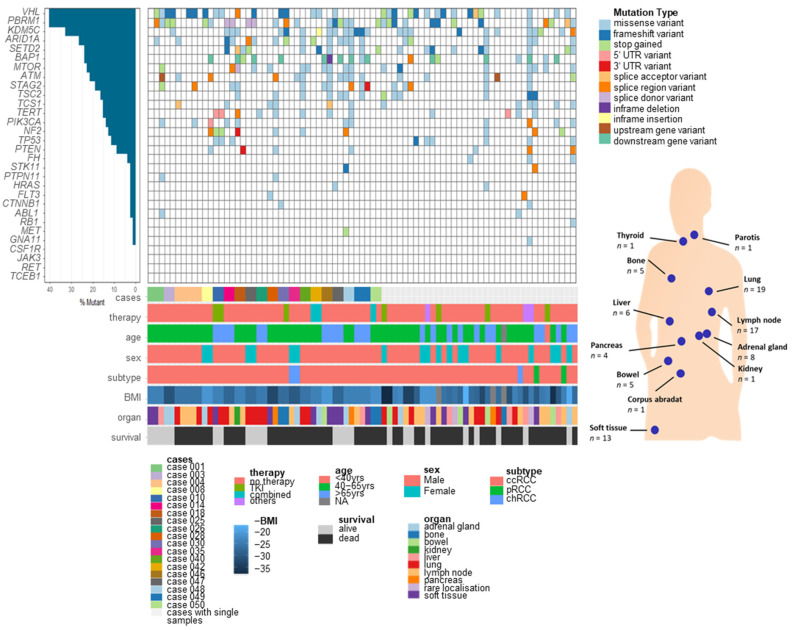
Somatic variants in metastasis samples of primary RCC analysed through NGS panel approach. Two metastasis samples were excluded from variant analysis due to a hypermutated genetic landscape, resulting in a final cohort of 79 samples from 55 patients. Frequency distribution, including information about mutation types in selected panel genes and patient information (cases with multiple metastases, therapy, patient’s age, sex, subtype of primary tumour, BMI (kg/m^2^), site of metastases, and survival), is shown.

The study cohort comprises 56 patients treated at the Department of Urology, University Hospital Tuebingen, Tuebingen, Germany (Table S1). Most of the patients (*n* = 52) presented with ccRCC, the main subtype of RCC. From these patients, 81 metastases (Table 1 and Table S1) were surgically resected and from 19 patients, multiple metastases were included. Further information about patients’ characteristics and metastasis specimen is given in Table S1. Metastasis samples have been investigated by targeted NGS of 32 cancer-related and known RCC driver genes selected as described in supplementary methods. Two metastasis samples were excluded from variant analysis due to a hypermutated genetic landscape, resulting in a final cohort of 79 samples from 55 patients. For further analyses, single nucleotide variants (SNV) and small indels were considered (for details, see supplementary data). Overall, *VHL* (40.5%), *PBRM1* (40.5%), and *KDM5C* (32.9%) were identified as most frequently mutated genes (Figure 1) in our cohort.

**Figure 2:**
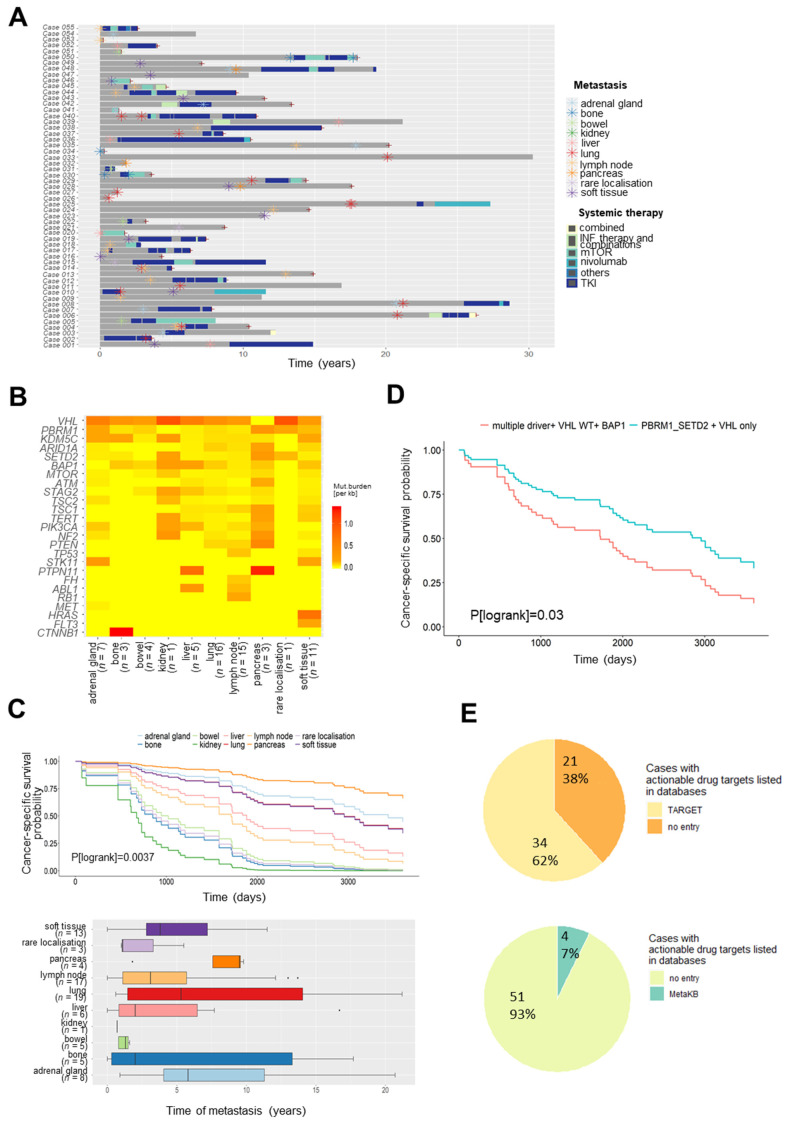
Somatic variants of RCC metastases in different metastatic sites. (**A**): Treatment timeline indicating course of therapy and patient’s survival. Time of surgical resection of metastasis is marked by asterisks. (**B**): Heatmap showing low (yellow) and high (red) mutational burden for each gene and organ for metastasis (*n* = 68) of cases without prior systemic therapy. Mean mutational load is displayed. (**C**): Association of the site of metastases with patient’s survival (*p*(logrank) = 0.00369) (upper panel). Time of occurrence of metastases in different organs after surgery of primary tumours is shown in the lower panel. (**D**): Kaplan–Meier plot showing the association of the somatic mutational composition and cancer-specific survival in our cohort (*p*(logrank) = 0.0334). Metastases (*n* = 32) harbouring multiple somatic drivers and *VHL* wildtype alleles were compared to *PBRM1*, *SETD2,* and *VHL* monodrivers (*n* = 47). (**E**): Mapping of somatic mutational events in our cohort to drug target information (TARGET drug recommendation, https://software.broadinstitute.org/cancer/cga/target, accessed on 15 March 2021) and to data on clinical significance using the VICC Meta-Knowledgebase (MetaKB). Pie plots indicate number of cases with recommendations in either of the databases.

The investigated metastases occurred in 18 different sites (Table S1), which were combined to ten organ groups. As shown in Figure 2A and Table S2, most of the cohort’s metastases (84%, *n* = 68) were surgically removed before treatment with any systemic therapy. For subsequent organ-specific analyses, we considered only these untreated metastases. Here, notable differences were found between organs (Figure 2B). The highest mutational burden in distant metastases was identified in the pancreas followed by the liver (Figure 2B, Figure S1), whereas the lowest mutational load was found in metastases of the bowel. Exclusion of patient samples from rare RCC subtypes (pRCC, chRCC) did not notably change results (data not shown).

### 3.2. Somatic Variants in Metastases for Prediction of Survival and Personalized Therapy

Correlation of clinical data revealed a significant association of the site of metastases with cancer-specific survival (Figure 2C, *p*(logrank) = 0.0034). Higher survival probability was observed for patients with metastases in the pancreas despite its high overall mutational burden. Additionally, 3 out of 4 pancreatic metastases in our cohort occurred >5 yrs after surgery of primary tumours, whereas metastases of the bowel developed within 1.6 yrs (Figure 2C).

As proposed by Turajlic et al. [10], progression in RCC might be influenced by the somatic mutational composition. In line, detailed survival analysis in our cohort indicated significantly worse cancer-specific survival probability for patients with metastases harbouring multiple somatic drivers and *VHL* wildtype alleles compared to *PBRM1*, *SETD2*, and *VHL* monodrivers (*p* (logrank) = 0.03) (Figure 2D). Here, metastases (*n* = 32) harbouring multiple somatic drivers and *VHL* wildtype alleles were compared to *PBRM1*, *SETD2,* and *VHL* monodrivers (*n* = 47), using Cox proportional hazards model with consideration of the partially multiple metastases per patient. The same trend was observed in only lung metastases (*p* (logrank) = 0.063), but survival analysis for metastases at different sites is limited because of small sample sizes per organ. 

Somatic mutations are not only important for prediction of patient’s outcome, but also enable stratification of patients towards therapies that either have been approved or are part of current clinical trials. Therefore, we mapped somatic mutation events in our cohort to drug target information (TARGET drug recommendation, https://software.broadinstitute.org/cancer/cga/target, accessed on 15 March 2021) and evaluated their clinical significance using the VICC Meta-Knowledgebase (MetaKB, Table S3), which provides summarized data from six different knowledgebases [26]. Thereby, we aimed to identify mutations in genes for which targeted therapies are already available or even recommended. Taken together, in 34 patients (62%) of our cohort, at least one drug target gene was listed. Clinically relevant and potentially “drug-able” mutations with specific recommendations in the Meta-Knowledgebase were found in 4 cases (7%) (Figure 2E, Table S3). 

### 3.3. Evolution of Somatic Variants in Recurrent Metastases over Time and Therapeutic Course

**Figure 3:**
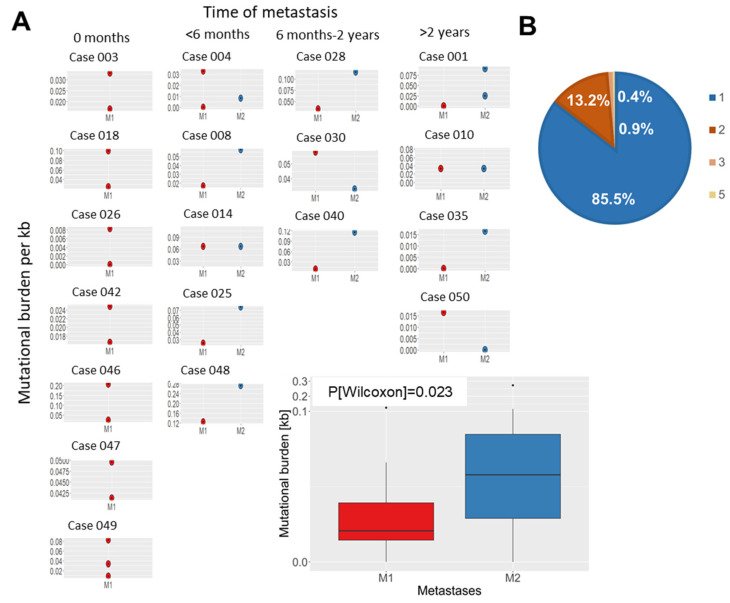
Somatic variants in recurrent metastases over time. (**A**): Mutational burden in synchronously resected and/or recurrent metastases. Cases are grouped according to timespan between metastases resections (0 months: synchronous resection of metastases; <6 months, 6 months–2 yrs, >2 yrs: timespan between resection of different metastases from the same patient). Mutational burden in recurrent metastases increased significantly over time (analysed by Wilcoxon signed-rank test; *p*(Wilcoxon) = 0.023) between the first metastases and the later ones. (**B**): Pie plot indicates the number of shared variants in metastases resected from the same patient.

Of note, for 19 patients at least two different metastasis samples from either one or more organ sites and/or time-points were available. Mutational burden in metastases that were surgically removed at the same time varied considerably in certain cases (Figure 3A). Analyses of recurrent metastases revealed higher mutational burden in most of the later metastases compared to earlier ones (Figure 3A).

**Figure 4:**
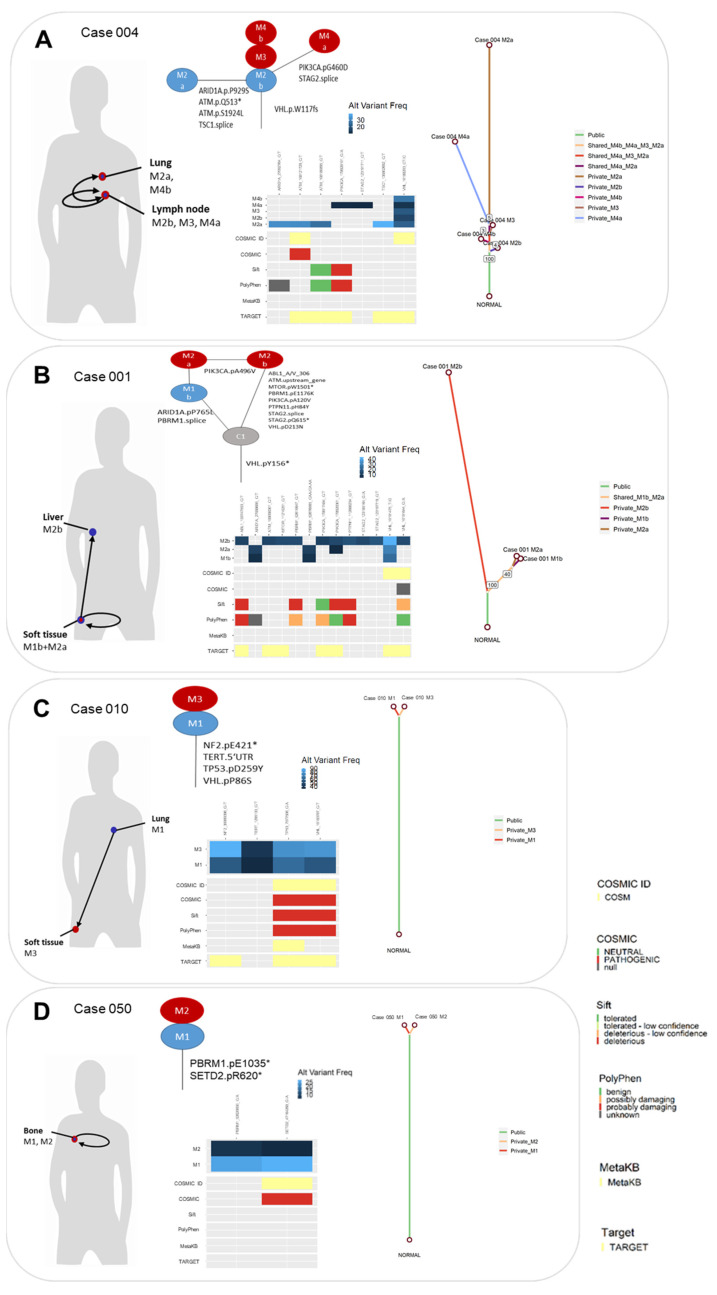
Somatic mutations in synchronously resected and/or metachronous metastases of individual patients ((**A**): case 004, (**B**): case 001, (**C**): case 010, and (**D**): case 050). Functional annotation of somatic variants using SIFT and PolyPhen, as well as COSMIC, MetaKB, and TARGET annotation is displayed. Phylogenetic trees of each case were constructed using MesKit [25]. Branches are coloured according to the distribution of mutations in different metastases. Lengths of the branches are proportional to the number of mutations. Support values of internal nodes are annotated within trees.

Overall, the mutational burden in recurrent metastases (Figure 3A) increased significantly over time (*p* = 0.023) between the first metastases and the later ones. As shown in Figure 3B, metastases from the same patient share up to five overlapping somatic mutations, indicating a common ancestor clone in most cases. Among all 12 cases with recurrent metastases, 13% of mutations (23/177) were also detected in the later metastases (Figure 4 and Figure S2). For instance, one patient (case 004, Figure 4A) developed multiple metastases in the lung and lymph nodes, which were surgically removed in three interventions within six months without prior systemic therapy. All metastases from this patient shared one somatic variant in *VHL*, suggesting a common clone of origin. 

The same was found in recurrent metastases, which were surgically resected in one patient after a time-period of >2 yrs (case 001, Figure 4B). In this case, the later metastasis in the same organ had more mutations in common with the original one than later metastasis in a different organ. Moreover, several other cases demonstrate more identical somatic events in recurrent metastases than private mutations even in metastases, which were resected more than 2 yrs later. For instance, in case 010 (Figure 4C) all four somatic mutations detected first in a lung metastasis were identified in an additional metastasis in the soft tissue removed >2 yrs later, indicating that key mutations might spread to metastatic sites during development of metastases. One of these shared somatic events (*TP53* D259Y; c.775G>T) is already listed in MetaKB with drug label information (Table S3). The same holds true for case 050 (Figure 4D), with two somatic events detected first in a bone metastasis and >2 yrs later in an additional metastasis in the bone removed after several treatment regimens. Of note, the detected variant allele frequency of the mutations in the second metastasis was lower, perhaps due to lower tumour extent. 

To investigate whether mutations shared in metastases are already present in primary tumours, we performed targeted sequencing in six cases with available matched primary tissue. Here, mutations were detected already in primary tumours of four cases (Table S4).

Based on our results, targeted therapy to metastases in one site most likely affects subsequent progression of the disease. Therefore, we next investigated somatic mutations in recurrent disease during therapeutic intervention to identify molecular mechanisms of drug resistance. In-depth analyses of the course of the disease and treatment in cases with at least two metastases indicated that some patients for which mTOR therapy was recommended (based on TARGET prediction) actually received everolimus/temsirolimus (Tables S2 and S3). However, not all of these patients seem to respond to mTOR therapy due to different reasons (Table S2). For instance, targetable mutations occurred only in selected metastases of an individual patient (e.g., case 018). Moreover, in addition to pharmacodynamic somatic targets, other mechanisms such as drug metabolism or drug transport contributing to intracellular drug concentrations in the tumour might be responsible for therapy failure or resistance [27,28]. As mTOR inhibitors and TKIs are in part substrates of *CYP3A5* and *ABCB1*, we genotyped as an example relevant *CYP3A5* and *ABCB1* variants in these patients (Table S5), indicating the presence of genetic variants (e.g., CYP3A5*1/*3 genotype in case 040) with functional consequences on drug metabolism or transport.

## 4. Discussion

Since metastasis is the main cause of cancer-related death in RCC, it is particularly important to understand the genetic landscape of metastases. In the present study, we studied 81 metastases surgically resected from 56 patients, including multiple metastases from 19 patients, which allows investigation of metastasis evolution over time and course of treatment. First, we established a novel gene panel for in-depth sequencing of 32 genes with impact on RCC development and progression. Of note, known driver genes identified in large-scale studies of primary tumours were included [6]. Compared to current whole-exome or whole-genome sequencing approaches, our panel approach enables sequencing to a higher depth (1000×), allowing identification of rare variants, which is particularly important for analyses of shared variants between different metastases from one individual. Overall, the frequency distribution of somatic mutations detected in our cohort is comparable to large-scale studies of primary tumours [6] with *VHL* (40.5%), *PBRM1* (40.5%), and *KDM5C* (32.9%) being the most frequently mutated genes in our cohort. Thus, the general genomic landscape of RCC seems to remain quite stable in metastasis.

Further analyses considering the different metastatic sites in our cohort revealed the highest mutational burden in distant metastases of the pancreas followed by the liver, whereas lowest mutational load was found in metastases of the bowel. Despite the high overall mutational burden, survival probability for patients with metastases in the pancreas was higher in our cohort. Additionally, most pancreatic metastases in our cohort occurred relatively late (>5 yrs) after surgery of primary tumours compared to those of the bowel (within 1.6 yrs). Recently, the higher survival rates in pancreatic metastases were associated with angiogenesis and an uninflamed stroma, which most likely results in increased response to antiangiogenic therapies but, on the other hand, resistance to immune checkpoint therapy [29]. 

The importance of the mutational composition of primary RCC tumours for disease progression and metastasis was recently investigated by Turajlic et al. [10], who proposed different genetic ccRCC subtypes. For instance, multiple *PBRM1*-driven and *VHL* monodriver subtypes predominately progress to a solitary metastatic site, whereas ccRCC tumours with multiple clonal drivers, *BAP1*-driven, and *VHL* wildtype subtypes show rapid progression to multiple sites [11,30]. Association of the mutational composition in metastases and cancer-specific survival in our cohort indicated significantly worse cancer-specific survival probability for patients with metastases harbouring multiple somatic drivers and *VHL* wildtype alleles compared to *PBRM1-*, *SETD2-,* and *VHL* monodrivers. Thus, our data derived from sequencing of metastasis are in line with results from primary ccRCC. Although we observed a trend that the same association is true in lung metastases only, further analysis considering mutational composition at different metastatic sites is limited because of small sample sizes per organ group.

It is increasingly recognized that somatic mutations are not only valuable predictors of a patient’s outcome, but also allow patient stratification towards therapies. Mapping of somatic events to drug target information (TARGET drug recommendation, https://software.broadinstitute.org/cancer/cga/target, accessed on 15 March 2021) indicated that at least one drug target gene was mutated in 62% of cases and clinically relevant mutations with specific recommendations in the Meta-Knowledgebase [26] have even been found in 7%. Although our panel was not designed to cover potentially actionable genes, our data indicate that sequencing of metastasis offers the potential to support disease management.

In order to improve treatment and disease management in RCC, deciphering the evolutionary development of metastatic disease is crucial. We therefore investigated for 19 patients at least two different metastasis samples from either one or more organ sites and/or time points. Interestingly, the mutational burden in recurrent metastases increased significantly over time, and recurrent metastases displayed shared mutations to earlier ones indicating common ancestor clones in most cases. Several cases even demonstrate more identical somatic events in recurrent metastases than private mutations even in metastases resected more than 2 yrs later and after several treatment regimens. Of note, whether the later metastases were seeded from the primary tumour or from earlier metastases would require additional mutational profiling of the primary tumours. Unfortunately, matched primary tumours were only available for a small subset of recurrently metastasized cases of our RCC cohort, since surgery of most primary tumours was not performed at our University Urology Department. In this subset, we confirmed the presence of shared variants in primary tumours of four out of six cases. Of course, the reason for a lack of mutations in the other two cases could be low variant frequency. Interestingly, case 001 presented with an additional tumour in the other kidney from which no tissue was available. Thus, it might be speculated that the metastases are derived from the bilateral tumour. In general, several competing models of tumour evolution, namely linear, branching, neutral, and punctuated, or even mixed models, are currently discussed [31]. Our findings show that cases share a set of mutations, which indicates a common evolutionary origin, but our approach does not enable us to resolve admixtures of clones. Single-cell sequencing approaches or monitoring circulating DNA would be required to reliably resolve the clonal architecture and evolution of metastases from our study. 

Based on our results, targeted therapy to metastases in one site most likely affects subsequent progression of disease with consequences for tissue biomarker-driven treatment strategies. Therefore, in-depth knowledge about somatic mutations in recurrent disease during therapeutic intervention enables identification of molecular mechanisms of drug resistance. However, in view of the different therapy regimens applied in patients with recurrent metastases in our cohort, identification of prognostic and predictive biomarkers was limited. For instance, only three cases with recurrent metastases actually received nivolumab. Thus, large-scale studies are warranted to investigate an association of mutational burden in metastases and the efficacy of currently applied checkpoint inhibitors. Of note, eight patients for whom mTOR therapy was recommended (based on TARGET prediction) in our cohort actually received everolimus/temsirolimus. Nevertheless, response in these patients to mTOR therapy was poor except in one patient, partly because targetable mutations did not occur in all metastases of an individual patient. Comparable to our results based on the study of metastases, no correlation was found in a recent study between rapalog therapy and somatic events in mTOR pathway genes in primary tumours of patients with metastatic RCC [32]. Since mTOR inhibitors and TKIs are, in part, substrates of drug metabolizing enzymes and drug transporters, germline variants in the respective genes might contribute to therapy failure or resistance as well [27,28,33]. Exemplarily, results from *CYP3A5* and *ABCB1* genotyping in our cohort indicates the presence of genetic variants with functional consequences, which might complement therapy selection in the future.

## 5. Conclusions

The somatic profile of RCC metastases revealed a high frequency of mutations in known RCC driver genes like VHL identified in primary RCC. Notably, in the majority of recurrent metastases, key mutations are shared between metastases even in different organs or after systemic therapies in later metastases. Although our sample set is small, it indicates that such shared variants frequently occur, thereby providing valuable information for personalized therapeutic management of recurrent disease.

## Figures and Tables

**Table 1:** Patient cohort.

Characteristics of Patients and Primary Tumours (*n* = 56)	Levels/Summary Statistics	No.	%
Sex	male	40	71.4
	female	16	28.6
Age (yrs) at diagnosis of primary RCC	median (range)	60.6 (29.2–77.5)	
T	1	12	21.4
	2	8	14.3
	3	29	51.8
	4	2	3.6
	na	5	8.9
N	0	44	78.6
	1	3	5.4
	2	3	5.4
	na	6	10.7
M	0	39	69.6
	1	12	21.4
	na	5	8.9
G	1	6	10.7
	2	27	48.2
	3	16	28.6
	na	7	12.5
Follow-up time (yrs) from date of diagnosis of primary RCC	median (range)	9.15 (0.2–30.3)	
Cancer-related death	no	19	33.9
	yes	37	66.1
**Characteristics of Metastasis/Local Recurrence Specimens (*n* = 81)**	**Levels/Summary Statistics**	**No.**	**%**
metastatic site	**Organ Group**		
	adrenal gland	8	9.9
	bone	5	6.2
	bowel	5	6.2
	local recurrence (kidney)	1	1.2
	liver	6	7.4
	lung	19	23.5
	lymph node	17	21.0
	pancreas	4	4.9
	rare localisation	3	3.7
	soft tissue	13	16.0
Age (yrs) at metastasis resection	median (range)	66.9 (31.6–80.6)	
Follow-up time (yrs) from date of metastasis resection	median (range)	5 (0–11.3)	
Systemic therapy before metastasis resection	no	68	84.0
	yes	13	16.0

## Data Availability

The datasets that support the findings of the present study are available from the corresponding author on reasonable request.

## Supplementary Materials

The following are available online at https://www.mdpi.com/article/10.3390/cancers13246221/s1, Table S1: Patient cohort; Table S2: Overview about resection of metastases and therapeutic intervention; Table S3: Overview about target drug information; Table S4: Somatic mutations in primary RCC of patients; Table S5: *ABCB1* and *CYP3A5* genotypes of selected cases; Figure S1: Mutational burden (per kb sequencing length) in different sites of metastasis for cases without prior systemic therapy; Figure S2: Somatic mutations in synchronously resected and/or metachronous metastases of individual patients. Functional annotation of somatic variants using SIFT and PolyPhen, as well as COSMIC, MetaKB and TARGET annotation is displayed. Phylogenetic trees of cases for which at least one mutation in each metastasis was detected were constructed using MesKit. Branches are coloured according to the distribution of mutations in different metastases. Lengths of the branches are proportional to the number of detected mutations. Support values of internal nodes are annotated within trees.
