# Supplementary material for: Characterization of Genetic Heterogeneity in Recurrent Metastases of Renal Cell Carcinoma
Source: Cancers (Basel). 2021 Dec 10;13(24):6221. doi: 10.3390/cancers13246221 (PMC8699544; doi:10.3390/cancers13246221)
Supplement: Supplementary file 1 [file cancers-13-06221-s001.zip › cancers-1471141-supplementary.pdf]

Supplementary Data

C. Sauter-Meyerhoff et al., Characterization of genetic heterogeneity in recurrent metastases of renal cell carcinoma

Supplementary material and methods, Tables S1-S5 and Figures S1-S2

Material & Methods

Patient cohort:

The study cohort comprises 56 patients treated at the Department of Urology, University Hospital Tuebingen, Tuebingen, Germany (Table S1). Median age at primary diagnosis was 60.6 years [range 29.2-77.5] with male predominance (n=40, female n=16). Most of the patients (n=52) were diagnosed with clear cell RCC (ccRCC), the main subtype of RCC, in addition to two papillary and chromophobe RCC cases, respectively. In total, 81 formalin fixed and paraffin embedded (FFPE) metastasis samples from these patients were collected after surgical intervention. Multiple metastases, including 6 matched primary tumours, were obtained from 19 patients. Metastases were diagnosed in 18 different organs which were combined to 10 organ groups (Figure 1A, Table S1). Use of the tissue was approved by the ethics committee of the University of Tuebingen and informed written consent was provided by each subject prior to surgical resection. Further information about patient's characteristics and collected metastasis samples is given in Table S1.

DNA Extraction

The surgical resected metastasis samples were formalin fixed and paraffin embedded (FFPE) and tumour regions were histologically defined by pathologists. DNA from these regions was extracted as previously described [1]. DNA concentration and quality was determined using Nanodrop and Qubit DNA High sensitivity kit.

Next generation sequencing (NGS)

Genetic analyses were performed through next-generation sequencing (NGS) using a newly established gene panel targeting gene regions of 32 different genes which are already known to play an important role in the development and progression of RCC.

Therefore, DNA sample quality was assessed using the TruSeq FFPE DNA Library Prep QC Kit (Illumina, San Diego, CA), a qPCR-based assay to determine the fragmentation status and the amplification potential. Dependent on QC results 10 to 100 ng DNA were used for library preparation as recommended.

Library preparation was performed using the TruSeq Custom Amplicon Low Input Library Prep Kit (Illumina, San Diego, CA) and a newly established custom gene panel (DesignStudio, Illumina, San Diego, California) targeting all exonic regions or selected gene regions of the following 32 genes which were already known to play an important role in the development and progression of RCC: *ABL1, ARID1A, ATM, BAP1, CSF1R, CTNNB1, FH, FLT3, GNA11, HRAS, JAK3, KDM5C, KMT2C/MLL3, MET, MTOR, MUC4, NF2, PBRM1, PIK3CA, PTEN, PTPN11, RB1, RET, SETD2, STAG2, STK11, TCEB1/ELOC, TERT, TP53, TSC1, TSC2, VHL.* The DNA samples were processed according to the manufacturer's instructions and uniquely indexed paired-end libraries were produced for each DNA sample with subsequent sequencing of pooled libraries on a MiniSeq platform (Illumina, San Diego, CA; mean coverage 1081.6). FastQ files including sequence quality scores were produced for further analyses.

NGS data analysis

Raw reads were mapped to the GRCh37/hg19 reference and amplicon primer sequences were soft-clipped by Local Run Manager (Illumina, San Diego, CA). Resulting alignments were examined for somatic variants by Illumina's Somatic Variant Caller (SVC, recommended for tumour samples) and LoFreq v2.1.5. [2] Sequence Pilot (SeqP) module SeqNext (JSI medical systems GmbH, Ettenheim, Germany) was used for read alignment, variant calling and annotation in parallel. The variants identified by the three variant callers were normalized using bcftools norm v1.11.

Finally, only variants found in all three variant datasets intersected by bcftools isec v1.11 were used for further analysis. We applied two different filter settings to this dataset. For high quality samples (Ct ≤ 3), variants with read depth > 200 and variant allele frequency ≥ 10% were kept; for low quality samples (Ct > 3), variants with read depth > 200 and variant allele frequency ≥ 25% were kept. The described analysis strategy was chosen to minimize the call of false positive variants due to sequencing artifacts. Limitations of this strategy were identified for variant calling in base repeat regions due to differences of variant position annotations between SVC and SeqP software. The sequenced gene regions of *MUC4* and *KMT2C* were excluded from the analysis due to known alignment/sequencing problems [3]. Two metastasis samples were excluded from variant analysis due to a hypermutated genetic landscape, resulting in a final patient cohort of 79 samples from 55 patients used for further analyses. Variants of these samples were annotated using the Illumina VariantStudio v3.0 data analysis software (Illumina, San Diego, CA) including information about dbSNP and COSMIC IDs. Annotations were

used to filter variants: Intronic and synonymous variants were removed. Germline variants were excluded by removing all variants present in the genome aggregation database for germline variants (gnomAD) [4]. Quality score was set to a minimum score of 30 for each variant to minimize false positive variant rates. For interpretation of pathogenicity of novel missense variants prediction tools (SIFT:tolerated-deleterious; PolyPhen: benign-damaging) were used. Information about effects and treatment implications of specific cancer gene alterations were included by annotations from the Variant Interpretation for Cancer Consortium (VICC) meta-knowledgebase (v1) [5]. The TARGET (tumour alterations relevant for genomics-driven therapy) [6] database was used for patient-specific analyses and therapy recommendations for variants in specific genes as well.

For organ-specific analysis only samples without prior systemic therapy until metastasis resection were considered (n=66).

Presence of somatic mutations in matched primary tumours was analyzed through Sanger sequencing or MALDI-TOF MS (Table S4). Primers are available upon request.

Genotyping of PGx variants

Selected samples of patients with multiple metastases were genotyped for five SNPs in *ABCB1* (rs1045642, rs1128503, rs2032582 G>A, rs2032582 G>T) and *CYP3A5* (rs776746) that are associated with metabolism and transport of targeted therapies. Genotyping was performed by TaqMan technology (Applied Biosystems) using the Sequence Detection System ABI PRISM 7900HT (Applied Biosystems) according to the manufacturer`s protocol.

Statistical analyses

Statistical analyses were performed in R (version 3.6.1) using additional packages from CRAN (http://cran.r-project.org) and from the Bioconductor software project (http://www.bioconductor.org, version 3.11): *survival* [7] and *survminer* [8]. For survival analyses, Cox proportional hazards model with consideration of the partially multiple metastases per patient was applied. The proportional hazards assumption was checked based on Schoenfeld residuals. Cancer-specific survival was defined as time from surgery to death or last date of follow-up if alive. Data for patients who died from other causes than RCC were considered censored at the time of death. One-sided Wilcoxon signed rank test was used for statistical testing of mutational burden in recurrent metastases (Fig.2B). Statistical significance was defined as p-value < 0.05.

References:

1. Horn H, Pott C, Kalla J, Dreyling M, Rosenwald A, Ott G, et al. A multiplex MALDI-TOF MS approach facilitates genotyping of DNA from formalin-fixed paraffin-embedded tumour specimens. Pharmacogenet Genomics. 2010;20:598–604. doi:10.1097/FPC.0b013e32833deb16.
2. Wilm A, Aw PPK, Bertrand D, Yeo GHT, Ong SH, Wong CH, et al. LoFreq: A sequence-quality aware, ultra-sensitive variant caller for uncovering cell-population heterogeneity from high-throughput sequencing datasets. Nucleic Acids Res. 2012;40:11189–201. doi:10.1093/nar/gks918.
3. Shyr C, Tarailo-Graovac M, Gottlieb M, Lee JJY, van Karnebeek C, Wasserman WW. FLAGS, frequently mutated genes in public exomes. BMC Med Genomics. 2014;7:64. doi:10.1186/s12920-014-0064-y.
4. Karczewski KJ, Francioli LC, Tiao G, Cummings BB, Alföldi J, Wang Q, et al. The mutational constraint spectrum quantified from variation in 141,456 humans. Nature. 2020;581:434–43. doi:10.1038/s41586-020-2308-7.
5. Wagner AH, Walsh B, Mayfield G, Tamborero D, Sonkin D, Krysiak K, et al. A harmonized meta-knowledgebase of clinical interpretations of somatic genomic variants in cancer. Nat Genet. 2020;52:448–57. doi:10.1038/s41588-020-0603-8.

6. Van Allen EM, Wagle N, Stojanov P, Perrin DL, Cibulskis K, Marlow S, et al. Whole-exome sequencing and clinical interpretation of formalin-fixed, paraffin-embedded tumor samples to guide precision cancer medicine. Nat Med. 2014;20:682–8. doi:10.1038/nm.3559.
7. Therneau TM, Grambsch PM. Modeling Survival Data: Extending the Cox Model. Springer, New York. 2000.
8. Kassambara A, Kosinski M, Biecek P. survminer: Drawing Survival Curves using ‘ggplot2’. https://CRAN.R-project.org/package=survminer. 2020.

Table S1: Patient cohort

Characteristics of patients and primary tumours (n=56)	levels/summary statistics		no.	%
Sex	male		40	71.4
	female		16	28.6
Age (years) at diagnosis of primary RCC	median (range)		60.6 (29.2-77.5)	
T	1		12	21.4
	2		8	14.3
	3		29	51.8
	4		2	3.6
	na		5	8.9
N	0		44	78.6
	1		3	5.4
	2		3	5.4
	na		6	10.7
M	0		39	69.6
	1		12	21.4
	na		5	8.9
G	1		6	10.7
	2		27	48.2
	3		16	28.6
	na		7	12.5
Follow-up time (years) from date of diagnosis of primary RCC	median (range)		9.15 (0.2-30.3)	
Cancer-related death	no		19	33.9
	yes		37	66.1
Overall survival	alive		17	30.4
	dead		39	69.6
Metastasis	metachron		41	73.2
	synchron		14	25.0
	na		1	1.8
Subtype	ccRCC		52	92.9
	pRCC		2	3.6
	chRCC		2	3.6
**Characteristics of metastasis/local recurrence specimens (n = 81)**	**levels/summary statistics**		**no.**	**%**
Subtype	ccRCC		76	93.8
	pRCC		2	2.5
	chRCC		3	3.7
metastatic site	**organ group**	**site**		
	adrenal gland		8	9.9
	bone	lumbar vertebrae/spine, soft tissue osseous, femur condyles, deltoideus	5	6.2
	bowel	mesentery, mesocolon, jejunum, peritoneum, ileum	5	6.2
	local recurrence (kidney)		1	1.2
	liver		6	7.4
	lung	lung (lower, upper, central lobe), pleura	19	23.5
	lymph node	lymph nodes and mediastium	17	21.0
	pancreas		4	4.9
	rare localisation	corpus curetage, parotis, thyroid	3	3.7
	soft tissue	extremeties, muscular, abdominal	13	16.0

Age [years] at metastasis resection	median (range)	66.9 (31.6-80.6)	
Years from diagnosis of primary RCC to metastasis resection	median (range)	3.5 (0-21.2)	
Follow-up time (years) from date of metastasis resection	median (range)	5 (0-11.3)	
Systemic therapy before metastasis resection	no	68	84.0
	yes	13	16.0

Table S2: Overview about resection of metastases and therapeutic intervention.
Data are shown for all 55 patients included in NGS data analyses.

Case #	Metastases	Resection time points	Time between metastases occurrence	Subtype of primary tumour	Organ	Therapy at time of resection of first metastasis	Therapy sequence and outcome
Case 001	M1b, M2a, M2b	2	>2 years	ccRCC	soft tissue-soft tissue-liver	no therapy	Met-Met-Sun
Case 002	M2	1	NA	ccRCC	lung	TKI	Sun-Paz-Met-death
Case 003	M1a, M1b	1	NA	ccRCC	2xadrenal gland	no therapy	Met-Sun-Cabo
Case 004	M2a, M2b, M3, M4a, M4b	3	<6 months	ccRCC	lung, lymph node, lymph node, lymph node, lung	no therapy	Met-Met-Met-Sora-Paz-?-death
Case 005	M1	1	NA	ccRCC	bowel	no therapy	Met-Sora-Paz-Ever
Case 006	M2	1	NA	ccRCC	lung	no therapy	Met- Beva+INF alpha 2a-Axi-Sora-Sun-Lenva+Ever
Case 007	M1	1	NA	ccRCC	adrenal gland	no therapy	Met-Sun-Paz-death
Case 008	M1, M2	2	<6 months	ccRCC	adrenal gland, lung	no therapy	Met-Met-Paz-Nivo-Cabo-death
Case 009	M2	1	NA	ccRCC	lymph node	no therapy	Met
Case 010	M1, M3	2	>2 years	ccRCC	lung, soft tissue	TKI	Sun-Met-Met-Nivo
Case 011	M1	1	NA	ccRCC	lung	no therapy	Met
Case 012	M3	1	NA	ccRCC	lymph node	no therapy	Met-Sun-Paz-Sora-Ever-Axi-death
Case 013	M1	1	NA	ccRCC	lymph node	no therapy	Met-death
Case 014	M1, M2	2	<6 months	ccRCC	lung, lymph node	no therapy	Met-Met-Sun-death
Case 015	M2	1	NA	ccRCC	thyroid (rare loc.)	Radatio	Radatio-Met-Sun
Case 016	M1	1	NA	ccRCC	soft tissue	no therapy	Met-death
Case 017	M1	1	NA	ccRCC	lymph node	TKI	Sun-Met-Paz-Sun-Ever-Radatio-Sora-Axi-death
Case 018	M1a, M1b	1	NA	ccRCC	kidney, lymph node	no therapy	Met-Tem-Sun-death
Case 019	M1	1	NA	ccRCC	soft tissue	no therapy	Met-Sora-Sun-Beva+INF-Paz-death
Case 020	M1	1	NA	ccRCC	liver	no therapy	Met-Tem-death
Case 021	M1	1	NA	ccRCC	parotis (rare loc.)	no therapy	Met-death
Case 022	M1	1	NA	ccRCC	bowel	no therapy	Met-death
Case 023	M1	1	NA	ccRCC	soft tissue	no therapy	Met
Case 024	M1b	1	NA	ccRCC	lymph node	no therapy	Met-death
Case 025	M2, M3	2	<6 months	ccRCC	2xlung	no therapy	Met-Met-Paz-Sun-Radatio-Nivo
Case 026	M1a, M1b	1	NA	ccRCC	2xlung	no therapy	Met-death
Case 027	M1	1	NA	ccRCC	lung	no therapy	Met
Case 028	M1, M2	2	between 6 months and 2 years	ccRCC	soft tissue, pancreas	no therapy	Met-Met-Radatio-death

Case 029	M2	1	NA	ccRCC	lung	no therapy	Radatio-Met-Radatio-Sun-Paz-Ever-death
Case 030	M1, M2	2	between 6 months and 2 years	ccRCC	2xbone	no therapy	Met-Paz-Sora-Met-Tem-death
Case 031	M1	1	NA	ccRCC	bowel	TKI	Sun-Sora-Met-death
Case 032	M1	1	NA	ccRCC	pancreas	no therapy	Met-death
Case 033	M1	1	NA	ccRCC	lung	no therapy	Met
Case 034	M1	1	NA	ccRCC	bone	no therapy	Met-death
Case 035	M1, M2	2	>2 years	chRCC	lymph node, adrenal gland	no therapy	Met-Met-death
Case 036	M1	1	NA	ccRCC	liver	no therapy	Met-Sora-Sun-Nivo-Radatio-death
Case 037	M1	1	NA	ccRCC	liver	no therapy	Met-Sun-Ever-Paz-death
Case 038	M2	1	NA	chRCC	lymph node	no therapy	Met-Sun-death
Case 039	M2	1	NA	ccRCC	liver	INF	adj. INF/Il-Met
Case 040	M2, M3	2	between 6 months and 2 years	ccRCC	2xlung	no therapy	Met-Met-Sun-Ever-Sun-Paz-Sun-Radatio-Axi-Radatio-Radatio-death
Case 041	M1	1	NA	ccRCC	corpus curretage (rare loc.)	Tem	Met-Radatio-Tem-death
Case 042	M1a, M1b	1	NA	ccRCC	soft tissue, adrenal gland	INF + TKI	INF, IL+ 5FU-Sora-Met-death
Case 043	M3	1	NA	pRCC	soft tissue	no therapy	Met-Sun-death
Case 044	M1	1	NA	ccRCC	lymph node	no therapy	Met-Sun-Ever-Sora-Beva + INF-Paz-death
Case 045	M1	1	NA	ccRCC	lymph node	TKI	Sun-Met-Ever-Radatio-Sora-Beva + INF-death
Case 046	M1a, M1b	1	NA	ccRCC	bowel, soft tissue	no therapy	Met-Tem-death
Case 047	M2a, M2b	1	NA	ccRCC	2x soft tissue	no therapy	Met
Case 048	M1, M2	2	<6 months	ccRCC	adrenal gland, pancreas	no therapy	Met-Sun-Ever-Axi-Cabo
Case 049	M1a, M1b, M1c	1	NA	ccRCC	lymph node, liver, soft tissue	no therapy	Met-death
Case 050	M1, M2	2	>2 years	ccRCC	2x bone	no therapy	Met-Radatio-Sun-Sora-Ever-Paz-Radatio-Sun-Radatio-Tem
Case 051	M1	1	NA	ccRCC	bowel	no therapy	Met-Radatio-death
Case 052	M1	1	NA	ccRCC	liver	no therapy	Met-Sun-death
Case 053	M1	1	NA	pRCC	lymph node	no therapy	Met-death
Case 054	M1	1	NA	ccRCC	adrenal gland	no therapy	Met
Case 055	M1	1	NA	ccRCC	lymph node	no therapy	Met-Sun-Ever-Sora-Beva-Paz-death

Abbreviations: Met-metastasis resection, Beva-bevacizumab, INF-interferone, Axi-axitinib, Sora-sorafenib, Sun-sunitinib, Lenva-lenvatinib, Ever-everolimus, Tem-temsirolimus, Radatio-radiation, Paz-pazopanib, Cabo-cabozatinib, Nivo-nivolumab, TKI-tyrosine kinase inhibitor

Table S3: Overview about target drug information.

Case #	TARGET drug recommendation (https://software.broadinstitute.org/cancer/cga/target, 15.03.2021)	VICC Meta-Knowledgebase
Case 001	Imatinib, Dasatinib, Nilotinib, ABL1 inhibitors, PARP Inhibitors, Everolimus, Temsirolimus, PI3K/AKT/MTOR inhibitors	NA
Case 002	Everolimus, Temsirolimus, MTOR inhibitors	NA
Case 003	NA	NA
Case 004	PARP Inhibitors, PI3K/AKT/MTOR inhibitors, Everolimus, Temsirolimus	NA
Case 005	NA	NA
Case 006	Everolimus, Temsirolimus, MTOR inhibitors	NA
Case 007	PARP Inhibitors, Everolimus, Temsirolimus, MTOR inhibitors	NA
Case 008	PARP Inhibitors, HDAC Inhibitors, PI3K/AKT/MTOR inhibitors	NA
Case 009	NA	NA
Case 010	PI3K/AKT/MTOR inhibitors, Wee1 inhibitors, Chk1 inhibitors, kevetrin, APR-246, nutlins, gene therapy	Vemurafenib;Selumetinib;LY3009120
Case 011	HDAC Inhibitors, NA	NA
Case 012	Wee1 inhibitors, Chk1 inhibitors, kevetrin, APR-246, nutlins, gene therapy	NA
Case 013	NA	NA
Case 014	Everolimus, Temsirolimus, MTOR inhibitors, PI3K/AKT/MTOR inhibitors, Wee1 inhibitors, Chk1 inhibitors, kevetrin, APR-246, nutlins, gene therapy	NA
Case 015	PARP Inhibitors, Wee1 inhibitors, Chk1 inhibitors, kevetrin, APR-246, nutlins, gene therapy	NA
Case 016	HDAC Inhibitors, Wee1 inhibitors, Chk1 inhibitors, kevetrin, APR-246, nutlins, gene therapy	NA
Case 017	HDAC Inhibitors, NA	NA
Case 018	HDAC Inhibitors, Everolimus, Temsirolimus, PI3K/AKT/MTOR inhibitors, PARP inhibitors	NA
Case 019	NA	NA
Case 020	NA	NA
Case 021	NA	NA
Case 022	NA	NA
Case 023	NA	NA
Case 024	NA	NA
Case 025	HDAC Inhibitors, Everolimus, Temsirolimus, MTOR inhibitors	NA
Case 026	NA	NA
Case 027	PI3K/AKT/MTOR inhibitors, PARP inhibitors	NA
Case 028	PARP Inhibitors, Sunitinib, FLT3 inhibitors, Everolimus, Temsirolimus, MTOR inhibitors, PI3K/AKT/MTOR inhibitors	167869-21-8;Midorstaurin
Case 029	NA	NA
Case 030	HDAC Inhibitors, WNT inhibitors	NA
Case 031	PARP Inhibitors, HDAC Inhibitors, MAPK pathway inhibitors, Everolimus, Temsirolimus, PI3K/AKT/MTOR inhibitors, Wee1 inhibitors, Chk1 inhibitors, kevetrin, APR-246, nutlins, gene therapy	NA
Case 032	NA	NA
Case 033	PARP Inhibitors	NA

Case 034	NA	NA
Case 035	PARP Inhibitors	NA
Case 036	NA	NA
Case 037	NA	NA
Case 038	Imatinib, Dasatinib, Nilotinib, ABL1 inhibitors, HDAC Inhibitors, Everolimus, Temsirolimus, MTOR inhibitors	NA
Case 039	Sunitinib, FLT3 inhibitors	NA
Case 040	PARP Inhibitors, PARP Inhibitors, Everolimus, Temsirolimus, PI3K/AKT/MTOR inhibitors	NA
Case 041	PARP Inhibitors, HDAC Inhibitors, WNT inhibitors, MAPK pathway inhibitors, Everolimus, Temsirolimus PI3K/AKT/MTOR inhibitors, Wee1 inhibitors, Chk1 inhibitors, kevetrin, APR-246, nutlins, gene therapy	Everolimus+Letrozole;Sunitinib
Case 042	NA	NA
Case 043	Everolimus, Temsirolimus, PI3K/AKT/MTOR inhibitors, Dasatinib, src inhibitors, FAK inhibitors, Phenformin	NA
Case 044	NA	NA
Case 045	HDAC Inhibitors, Everolimus, Temsirolimus, MTOR inhibitors	NA
Case 046	PARP Inhibitors, HDAC Inhibitors, Everolimus, Temsirolimus, MTOR inhibitors	NA
Case 047	PARP Inhibitors, HDAC Inhibitors, MAPK pathway inhibitors, Everolimus, Temsirolimus, MTOR inhibitors	NA
Case 048	PARP Inhibitors, HDAC Inhibitors, Everolimus, Temsirolimus, PI3K/AKT/MTOR inhibitors, Dasatinib, src inhibitors, FAK inhibitors, Phenformin	NA
Case 049	PARP Inhibitors, PARP Inhibitors, HDAC Inhibitors, HDAC Inhibitors, HDAC Inhibitors, Everolimus, Temsirolimus, MTOR inhibitors	NA
Case 050	NA	NA
Case 051	NA	NA
Case 052	HDAC Inhibitors, PI3K/AKT/MTOR inhibitors, Everolimus, Temsirolimus	Buparlisib,Alpelisib+Fulvestrant,Serabelisib,Copanlisib,GDC,Taselisib
Case 053	PI3K/AKT/MTOR inhibitors	NA
Case 054	NA	NA
Case 055	PARP Inhibitors, Everolimus, Temsirolimus, PI3K/AKT/MTOR inhibitors, Wee1 inhibitors, Chk1 inhibitors, kevetrin, APR-246, nutlins, gene therapy	NA

Table S4: Somatic mutations in primary RCC of patients

Case #	Mutation	Confirmation in primary tumour	Method
case001	ARID1A_27088685_C/T	no	Sanger
	PBRM1_52676065_CAA/CAAA	N/A	
	PIK3CA_178928301_C/T	no	Sanger
	VHL_10191475_T/G	no	Sanger
case010	NF2_30069396_G/T	yes	MALDI-TOF MS
	TERT_1295133_C/T	N/A	
	TP53_7577506_C/A	yes	MALDI-TOF MS
	VHL_10183787_C/T	yes	Sanger
case014	PBRM1_52661288_C/A	yes	Sanger
	SETD2__47125410__GT/G	yes	Sanger
case018	BAP1_52439883_G/A	yes	Sanger
	VHL_10188195_T/G	yes	Sanger
case040	STAG2_123220435_C/T	no	Sanger
case047	PBRM1__52651548__C/CT	yes	Sanger
	VHL_10169309_T/C	yes	Sanger

N/A: no data available due to sequence context

Table S5: *ABCB1* and *CYP3A5* genotypes of selected cases

**Case #**	**Metastases**	***ABCB1* *rs1045642 C>T***	***ABCB1* *rs1128503 C>T***	***ABCB1* *rs2032582 G>T,A***	***CYP3A5* *rs776746***
Case 018	M1a, M1b	*T/T*	*T/T*	*T/T*	**3/*3*
Case 040	M2, M3	*T/T*	*T/T*	*T/T*	**1/*3*
Case 046	M1a, M1b	*C/T*	*C/T*	*G/T*	**3/*3*
Case 048	M1, M2	*C/T*	*C/C*	*G/G*	**3/*3*

Supplementary Figure S1

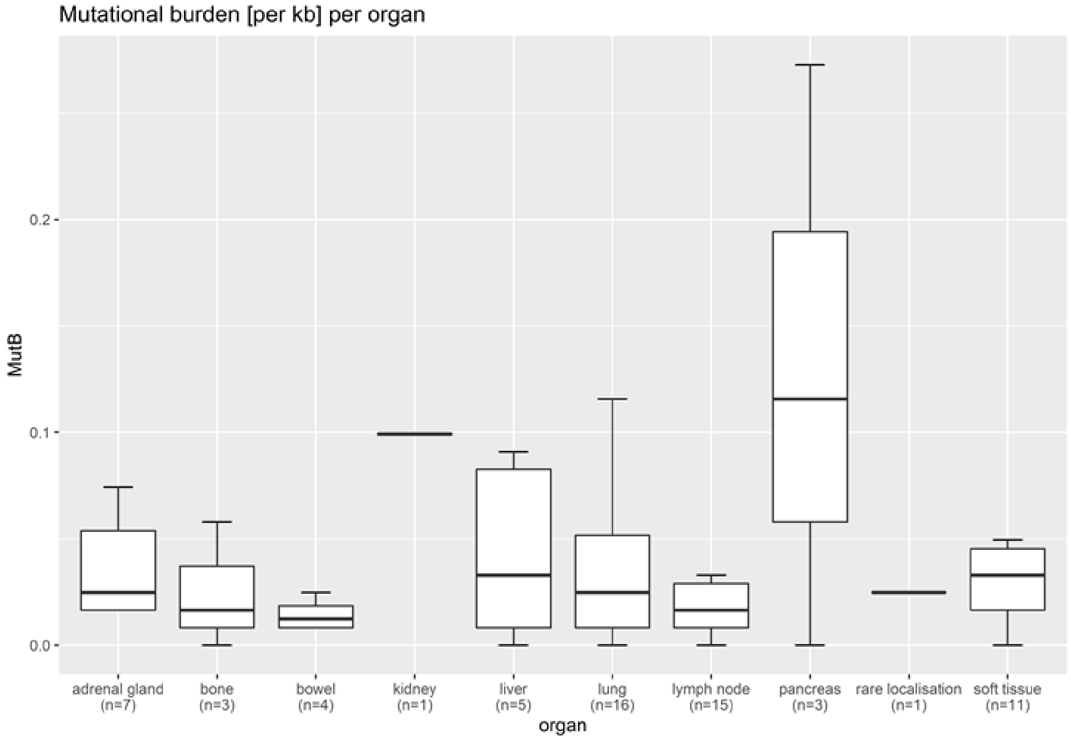

Figure S1: Mutational burden (per kb sequencing length) in different sites of metastasis for cases without prior systemic therapy.

Supplementary Figure S2

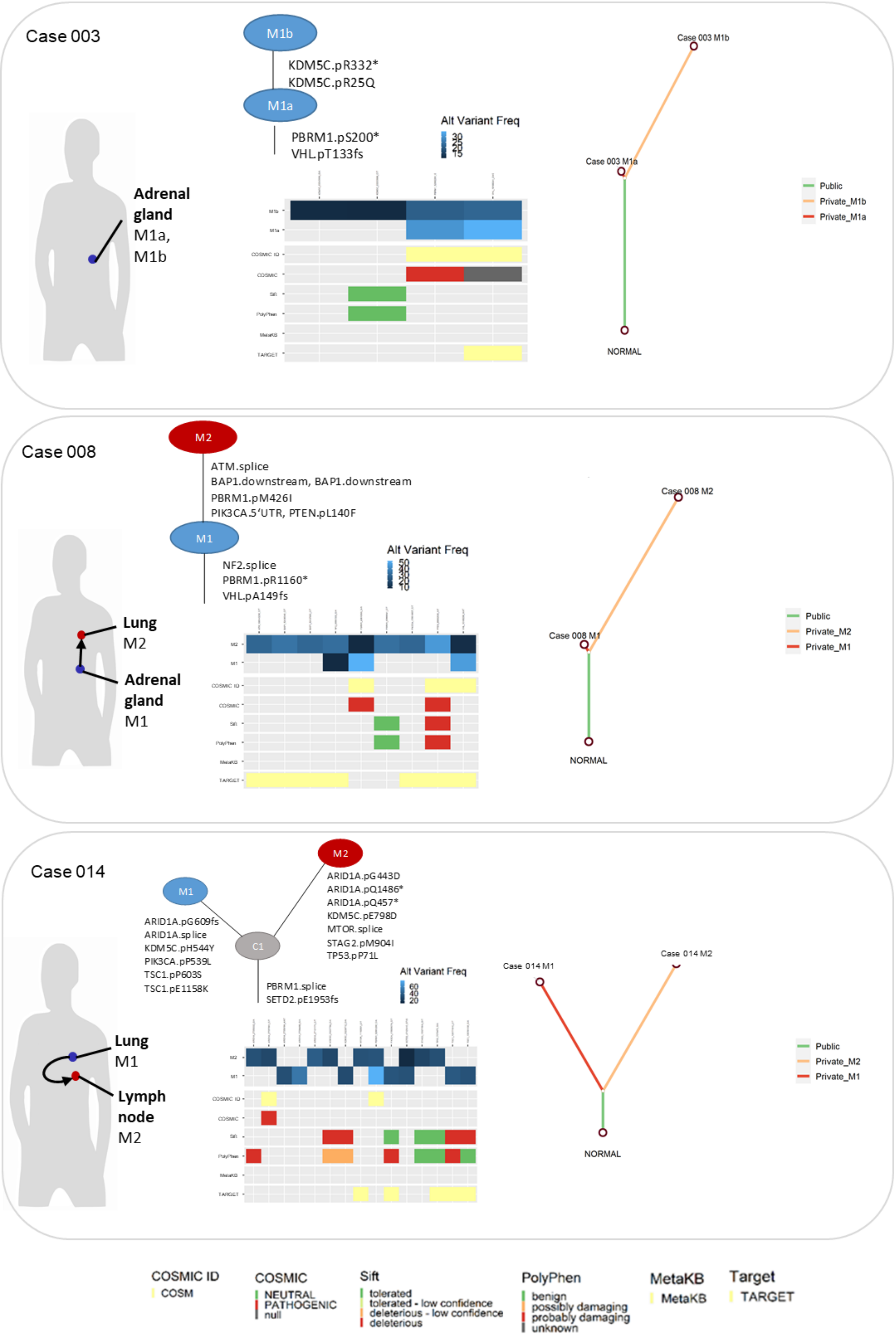

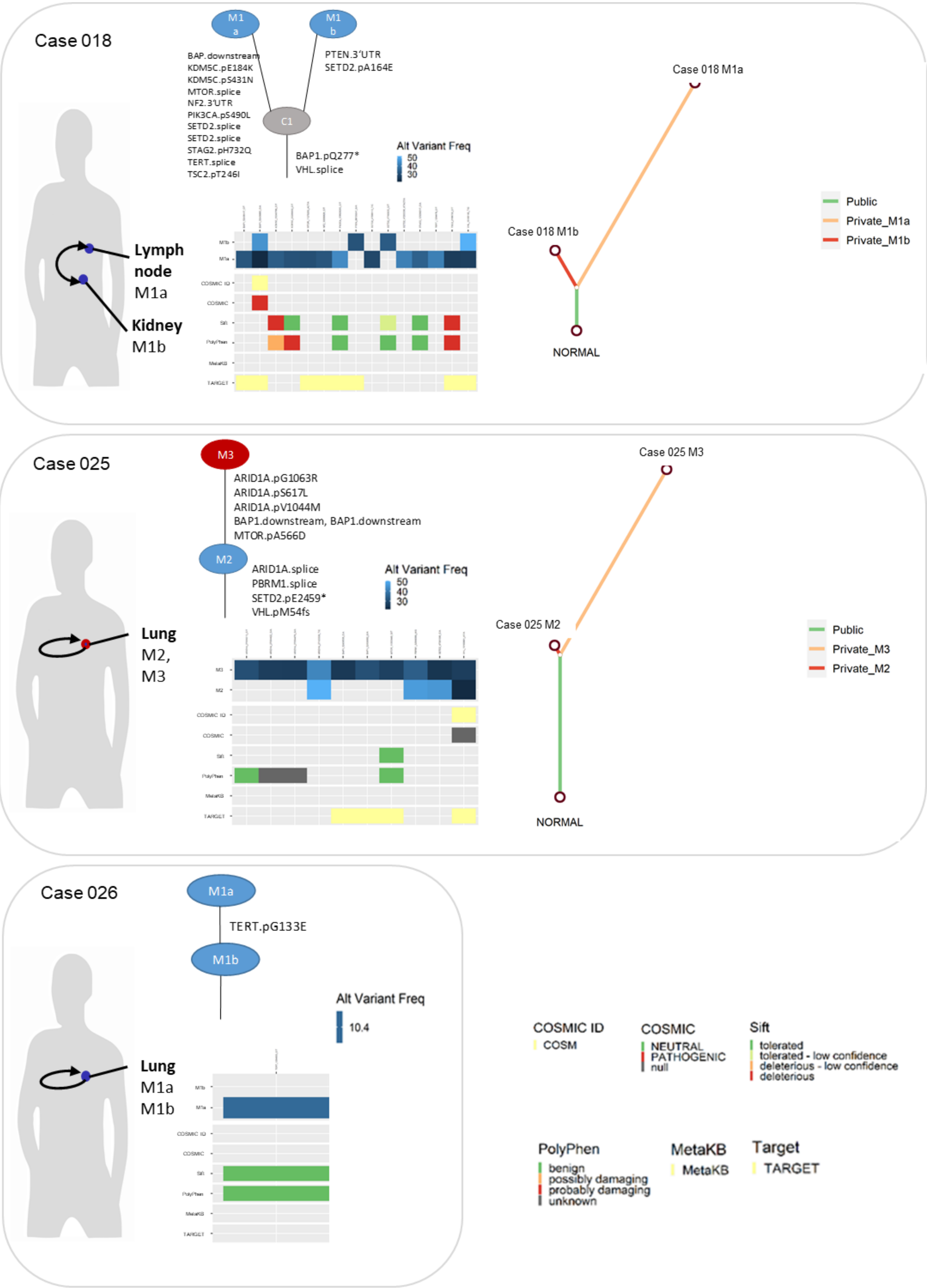
Case 018
M1a
M1b
BAP.downstream
KDM5C.pE184K
KDM5C.pS431N
MTOR.splice
NF2.3'UTR
PIK3CA.pS490L
SETD2.splice
SETD2.splice
STAG2.pH732Q
TERT.splice
TSC2.pT246I
PTEN.3'UTR
SETD2.pA164E
C1
BAP1.pQ277*
VHL.splice
Alt Variant Freq
50
40
30
Lymph node
M1a
Kidney
M1b
Case 018 M1a
Case 018 M1b
NORMAL
Public
Private_M1a
Private_M1b
Case 025
M3
ARID1A.pG1063R
ARID1A.pS617L
ARID1A.pV1044M
BAP1.downstream, BAP1.downstream
MTOR.pA566D
M2
ARID1A.splice
PBRM1.splice
SETD2.pE2459*
VHL.pM54fs
Alt Variant Freq
50
40
30
Lung
M2,
M3
Case 025 M3
Case 025 M2
NORMAL
Public
Private_M3
Private_M2
Case 026
M1a
TERT.pG133E
M1b
Alt Variant Freq
10.4
Lung
M1a
M1b
COSMIC ID
COSM
COSMIC
NEUTRAL
PATHOGENIC
null
Sift
tolerated
tolerated - low confidence
deleterious - low confidence
deleterious
PolyPhen
benign
possibly damaging
probably damaging
unknown
MetaKB
MetaKB
Target
TARGET

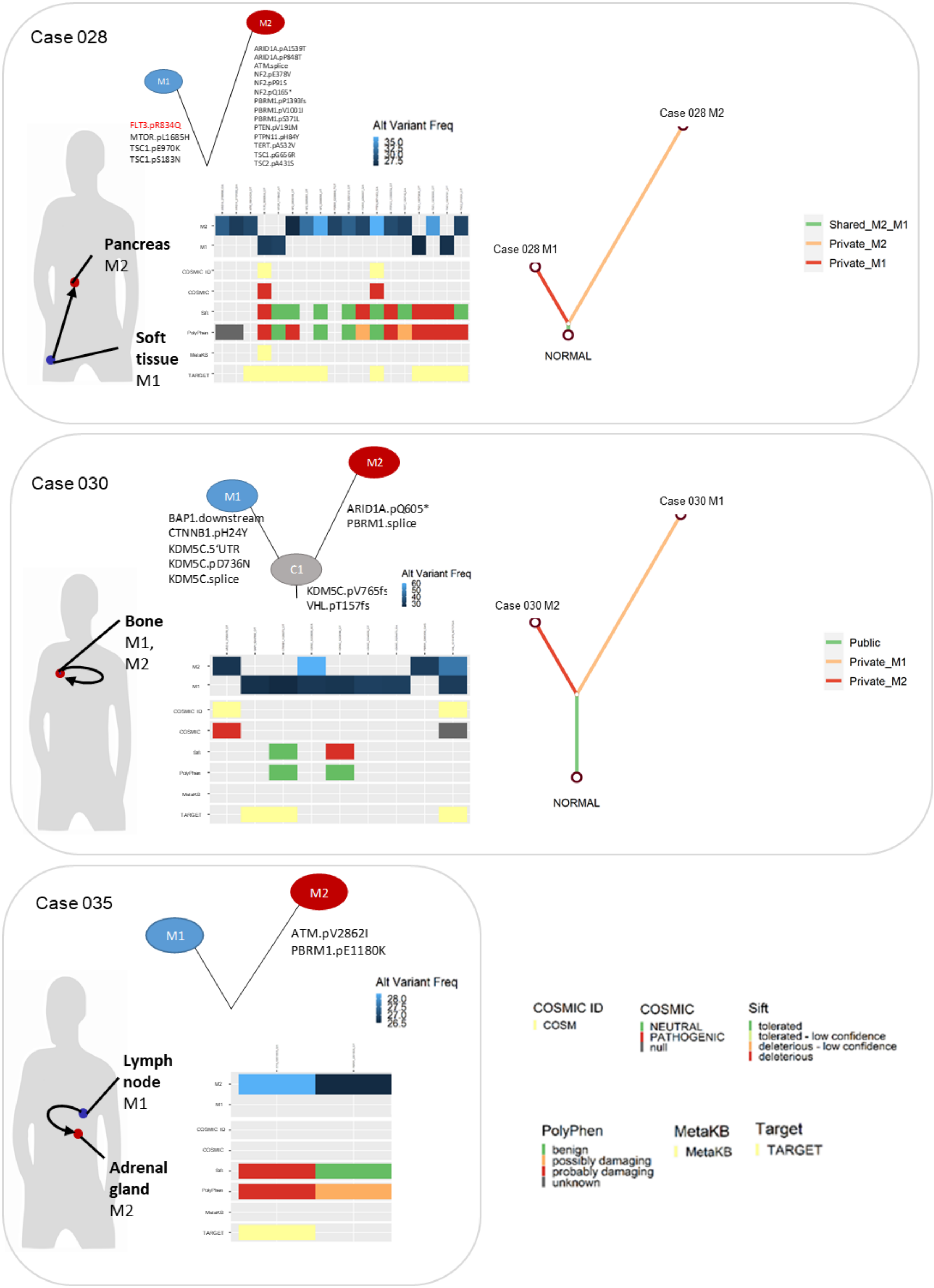

Case 028
M1
M2
FLT3.pR834Q
MTOR.pL1685H
TSC1.pE970K
TSC1.pS183N
ARID1A.pA1539T
ARID1A.pP848T
ATM.splice
NF2.pE378V
NF2.pP91S
NF2.pQ165*
PBRM1.pP1393fs
PBRM1.pV1001I
PBRM1.pS371L
PTEN.pV191M
PTPN11.pH84Y
TERT.pA532V
TSC1.pG656R
TSC2.pA431S
Alt Variant Freq
35.0
32.5
30.0
27.5
Pancreas
M2
Soft
tissue
M1
COSMIC ID
COSMIC
Sift
PolyPhen
MetaKB
TARGET
Case 028 M2
Case 028 M1
NORMAL
Shared_M2_M1
Private_M2
Private_M1
Case 030
M1
M2
C1
BAP1.downstream
CTNNB1.pH24Y
KDM5C.5'UTR
KDM5C.pD736N
KDM5C.splice
ARID1A.pQ605*
PBRM1.splice
KDM5C.pV765fs
VHL.pT157fs
Alt Variant Freq
60
50
40
30
Bone
M1,
M2
Case 030 M1
Case 030 M2
NORMAL
Public
Private_M1
Private_M2
Case 035
M1
M2
ATM.pV2862I
PBRM1.pE1180K
Alt Variant Freq
28.0
27.5
27.0
26.5
Lymph
node
M1
Adrenal
gland
M2
COSMIC ID
COSM
COSMIC
NEUTRAL
PATHOGENIC
null
Sift
tolerated
tolerated - low confidence
deleterious - low confidence
deleterious
PolyPhen
benign
possibly damaging
probably damaging
unknown
MetaKB
MetaKB
Target
TARGET

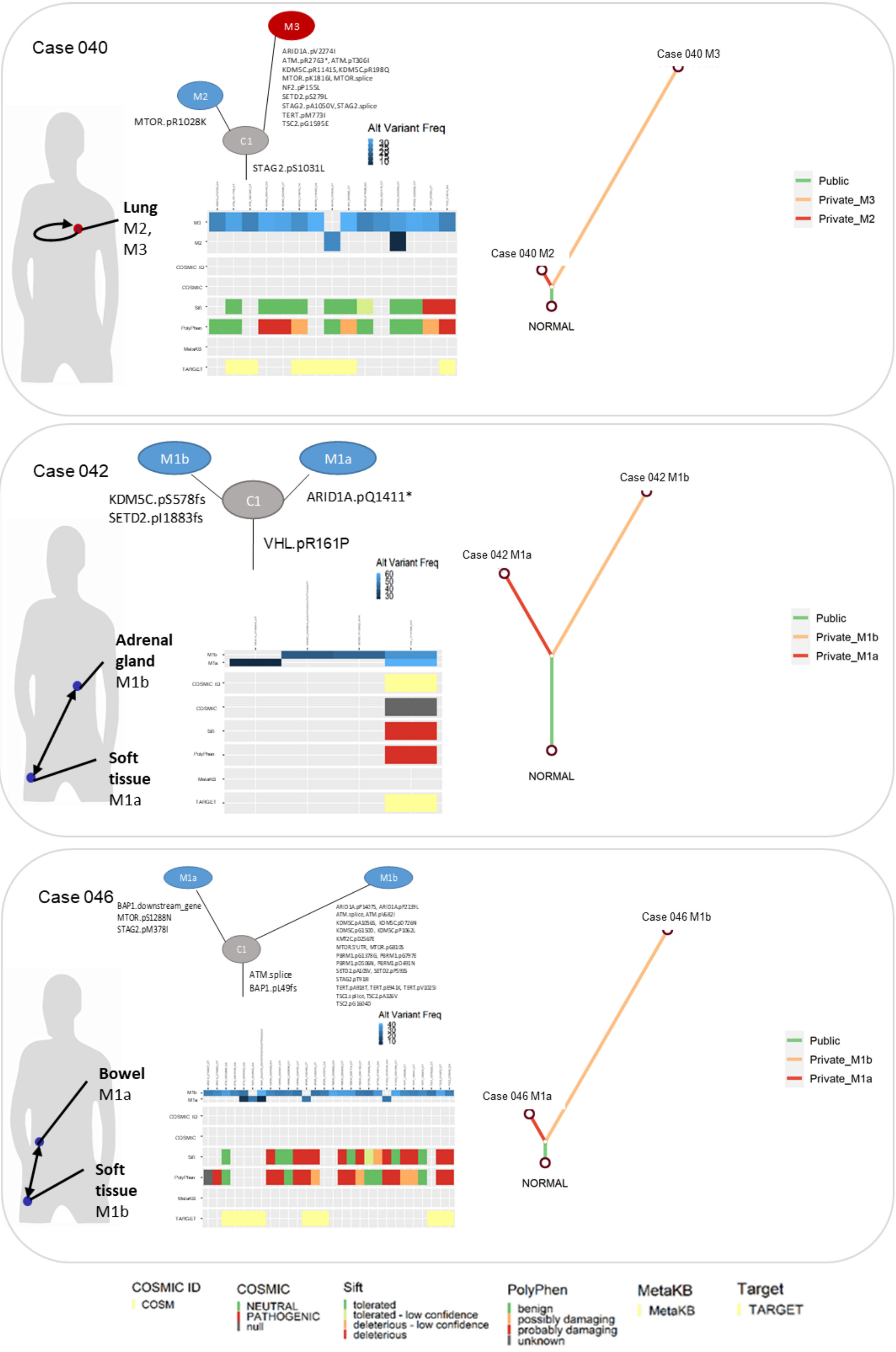

Case 040
M3
M2
C1
MTOR.pR1028K
ARID1A.pV2274I
ATM.pR2763*, ATM.pT306I
KDM5C.pR1141S,KDM5C.pR198Q
MTOR.pK1816I, MTOR.splice
NF2.pP155L
SETD2.pS279L
STAG2.pA1050V,STAG2.splice
TERT.pM773I
TSC2.pG1595E
STAG2.pS1031L
Alt Variant Freq
Lung
M2,
M3
Case 040 M3
Case 040 M2
NORMAL
Public
Private_M3
Private_M2
Case 042
M1b
M1a
C1
KDM5C.pS578fs
SETD2.pI1883fs
ARID1A.pQ1411*
VHL.pR161P
Alt Variant Freq
Adrenal gland
M1b
Soft tissue
M1a
Case 042 M1b
Case 042 M1a
NORMAL
Public
Private_M1b
Private_M1a
Case 046
M1a
M1b
C1
BAP1.downstream_gene
MTOR.pS1288N
STAG2.pM378I
ATM.splice
BAP1.pL49fs
Alt Variant Freq
Bowel
M1a
Soft tissue
M1b
Case 046 M1b
Case 046 M1a
NORMAL
Public
Private_M1b
Private_M1a
COSMIC ID
COSM
COSMIC
NEUTRAL
PATHOGENIC
null
Sift
tolerated
tolerated - low confidence
deleterious - low confidence
deleterious
PolyPhen
benign
possibly damaging
probably damaging
unknown
MetaKB
MetaKB
Target
TARGET

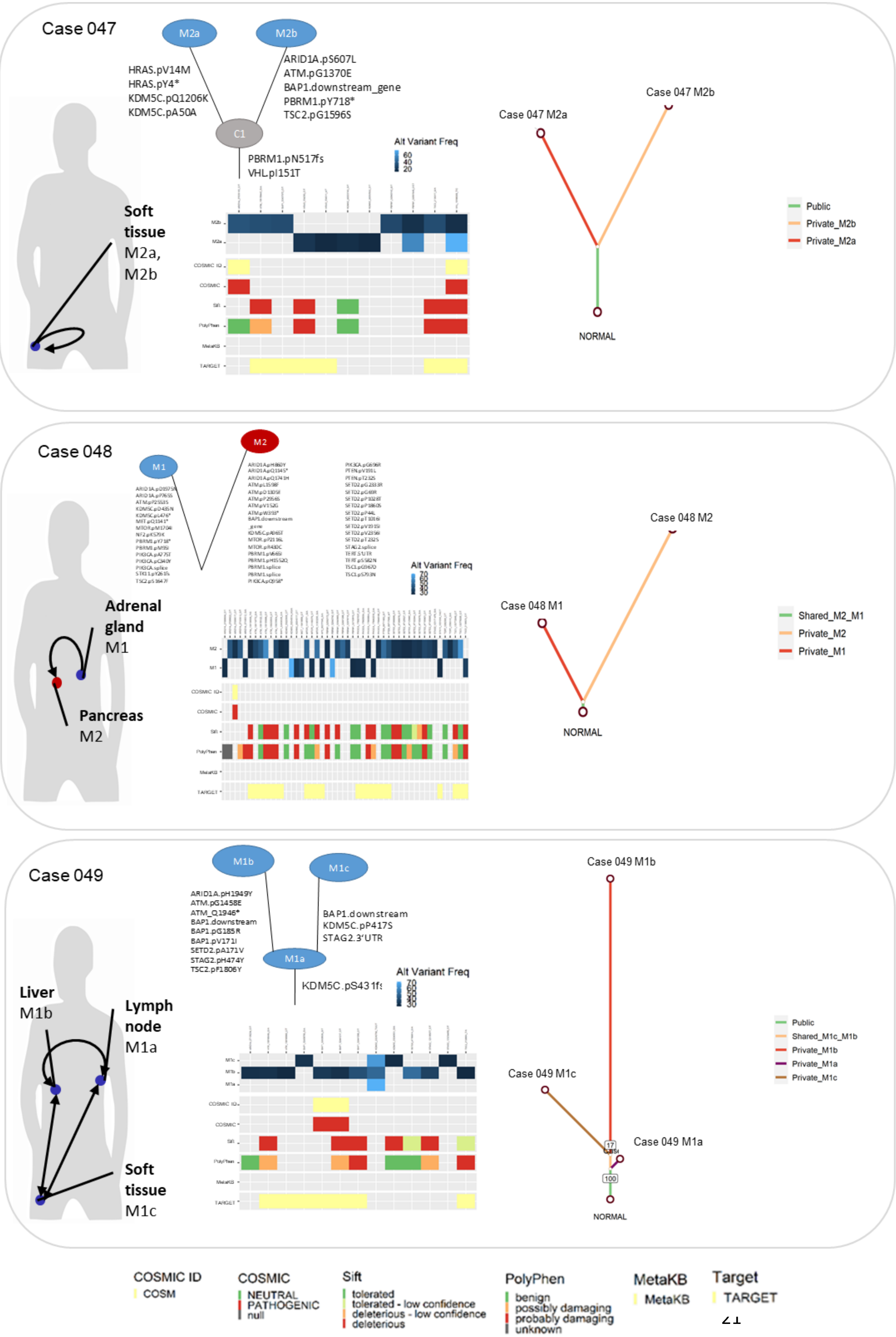
Case 047
M2a
M2b
C1
HRAS.pV14M
HRAS.pY4*
KDM5C.pQ1206K
KDM5C.pA50A
ARID1A.pS607L
ATM.pG1370E
BAP1.downstream_gene
PBRM1.pY718*
TSC2.pG1596S
PBRM1.pN517fs
VHL.pI151T
Alt Variant Freq
60
40
20
Soft tissue
M2a, M2b
Case 047 M2a
Case 047 M2b
NORMAL
Public
Private_M2b
Private_M2a
Case 048
M1
M2
Alt Variant Freq
Adrenal gland
M1
Pancreas
M2
Case 048 M1
Case 048 M2
NORMAL
Shared_M2_M1
Private_M2
Private_M1
Case 049
M1b
M1c
M1a
ARID1A.pH1949Y
ATM.pG1458E
ATM_Q1946*
BAP1.downstream
BAP1.pG185R
BAP1.pV171I
SETD2.pA171V
STAG2.pH474Y
TSC2.pF1806Y
BAP1.downstream
KDM5C.pP417S
STAG2.3'UTR
Alt Variant Freq
Liver
M1b
Lymph node
M1a
Soft tissue
M1c
Case 049 M1b
Case 049 M1c
Case 049 M1a
NORMAL
Public
Shared_M1c_M1b
Private_M1b
Private_M1a
Private_M1c
COSMIC ID
COSM
COSMIC
NEUTRAL
PATHOGENIC
null
Sift
tolerated
tolerated - low confidence
deleterious - low confidence
deleterious
PolyPhen
benign
possibly damaging
probably damaging
unknown
MetaKB
MetaKB
Target
TARGET

Figure S2: Somatic mutations in synchronously resected and/or metachronous metastases of individual patients. Functional annotation of somatic variants using SIFT and PolyPhen, as well as COSMIC, MetaKB and TARGET annotation is displayed. Phylogenetic trees of cases for which at least one mutation in each metastasis was detected were constructed using MesKit. Branches are coloured according to the distribution of mutations in different metastases. Lengths of the branches are proportional to the number of detected mutations. Support values of internal nodes are annotated within trees.
